# Rupture of urinary bladder secondary to bladder carcinoma with extensive abdominal gangrene: A case report

**DOI:** 10.1016/j.ijscr.2021.105717

**Published:** 2021-03-03

**Authors:** Mohamed Hafedh Saadi, Khaireddine Mrad Dali, Moez Rahoui, Ahmed Sellami, Sami Ben Rhouma, Yassine Nouira

**Affiliations:** Urology Department, Hopital La Rabta, Tunis, Tunisia

**Keywords:** Case report, Abdominal gangrene, Spontaneous rupture of urinary bladder, Bladder tumour

## Abstract

**Introduction and importance:**

Spontaneous rupture of the urinary bladder is rare but potentially severe. It is unusually related to bladder tumours. The morbidity and mortality rate are very high in these groups of patients.

**Case presentation:**

We present a case of a 62-year-old man who was known to have a bladder tumour who presented with extensive gangrene of the anterior abdominal wall. Imaging showed an extraperitoneal urinoma extended to the anterior abdominal wall secondary to a bladder rupture with posterior bladder wall thickening suggesting a bladder tumour. After optimization of the patient’s condition, urinoma drainage and upper urinary tract drainage by bilateral nephrostomy, excision of all necrotic tissues and a biopsy of the bladder lesion was performed. At a multidisciplinary meeting, we opted for a transurethral resection of the bladder followed by palliative chemotherapy considering that the tumour was locally advanced and depending on the disease course and patient’s condition.

**Clinical discussion:**

Gangrene secondary to urinary bladder rupture caused by transitional cell carcinomas is a very rare disease with poor oncological and infectious prognoses. For these reasons, treatment is often palliative.

**Conclusion:**

Urinary bladder rupture secondary to bladder carcinoma could rarely be complicated with abdominal gangrene. No standardized treatment is recommended seeing the extreme rarity of this disease and management should be discussed on a case-by-case basis.

## Highlights

• Spontaneous Urinary bladder rupture secondary to transitional cell carcinoma is a very rare entity.
• Abdominal gangrene is a severe necrotizing infection which is usually an extension of Fournier gangrene.
• Management of urinary bladder rupture with underlying transitional cell carcinoma of the bladder is not standardized.

## 1 Introduction

Rupture of the urinary bladder is usually associated with blunt or penetrating trauma, chronic diseases of the urinary bladder or bladder outflow obstruction [1]. Rupture of urinary bladder secondary to bladder tumour is extremely rare. The diagnosis of urinary bladder rupture may be difficult on account of the unreliable history and delayed and variable presentation [[1], [2], [3]]. We are reporting a case of a man presenting with gangrene of the anterior abdominal wall secondary to urinary bladder rupture. The work has been reported in line with the SCARE 2020 criteria [4].

## 2 Case report

**Fig. 1:**
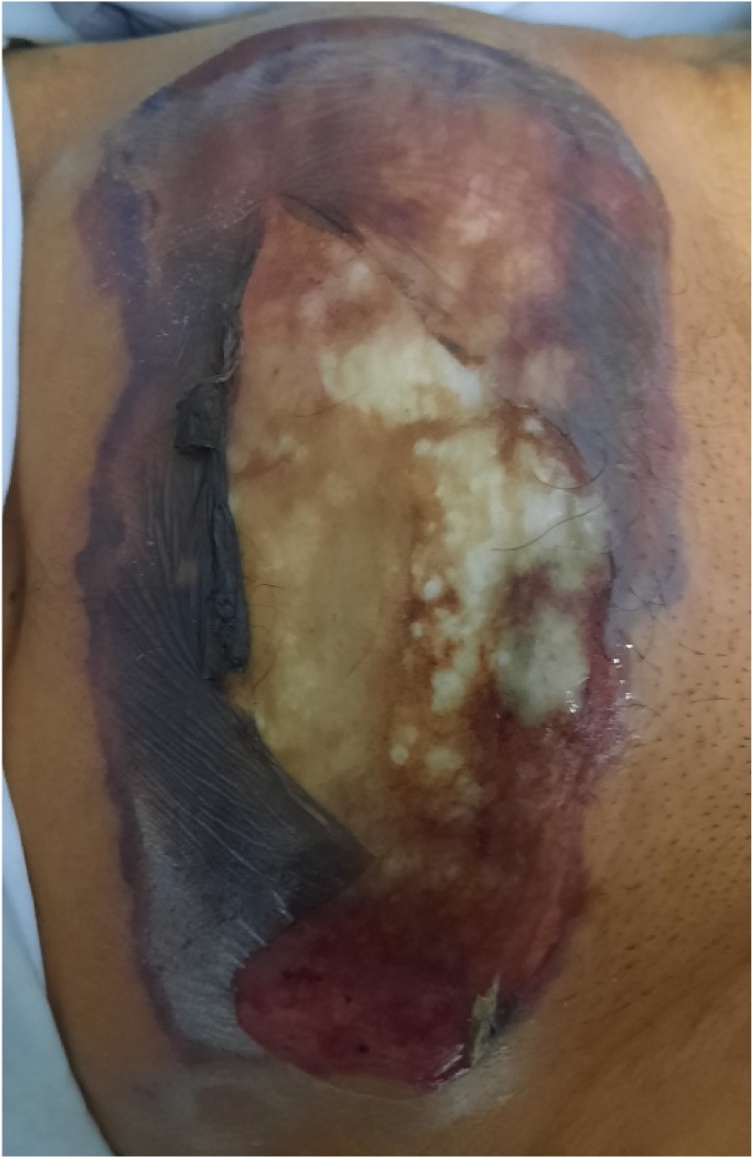
Gangrene of the anterior abdominal wall at presentation.

**Fig. 2:**
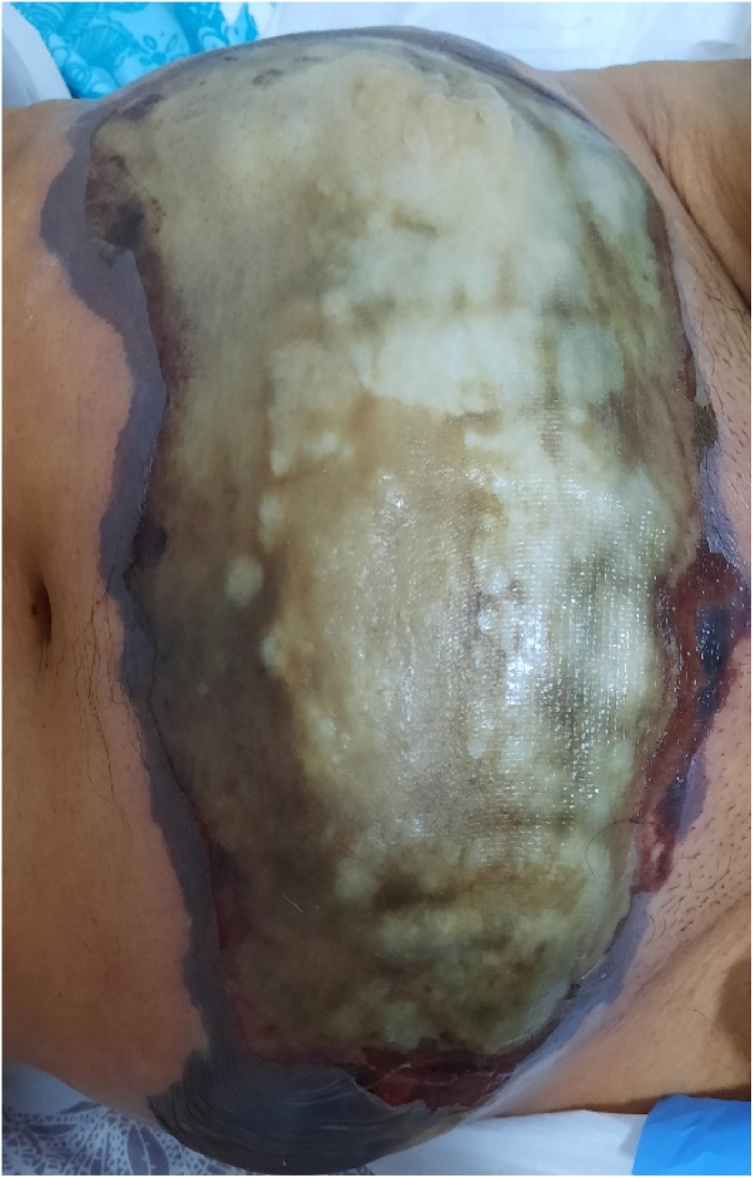
Extension of the gangrene one day after admission before urinary drainage and surgical excision.

**Fig. 3:**
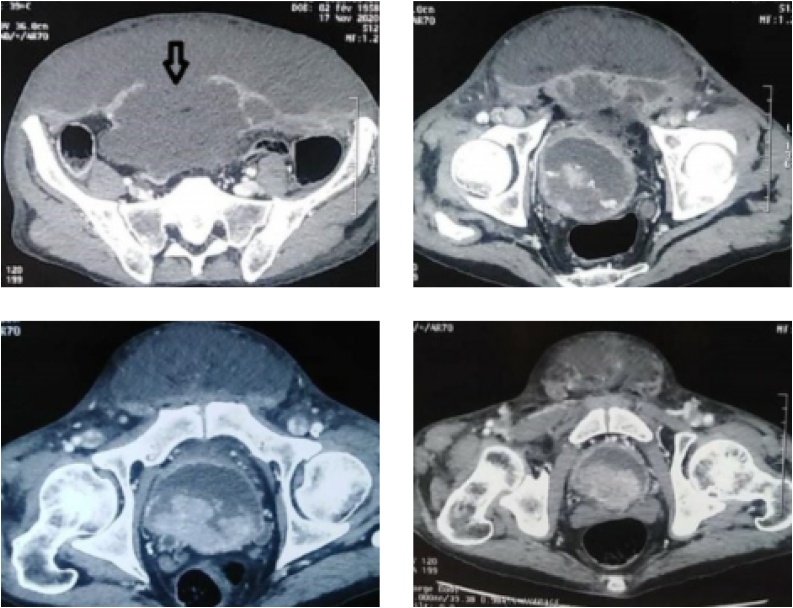
Initial CT scan showing bladder rupture on its anterior wall, large extraperitoneal urinoma and bladder tumour located on its posterior wall.

**Fig. 4:**
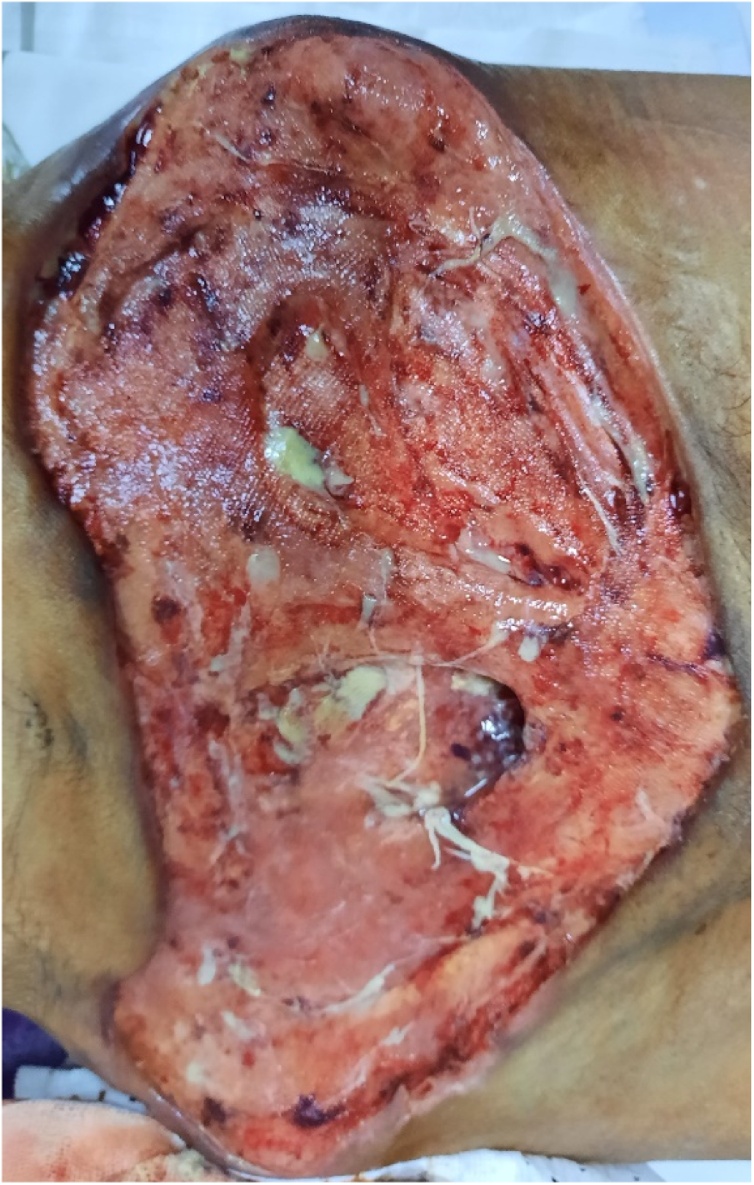
Excision necrotic tissue with large skin loss.

**Fig. 5:**
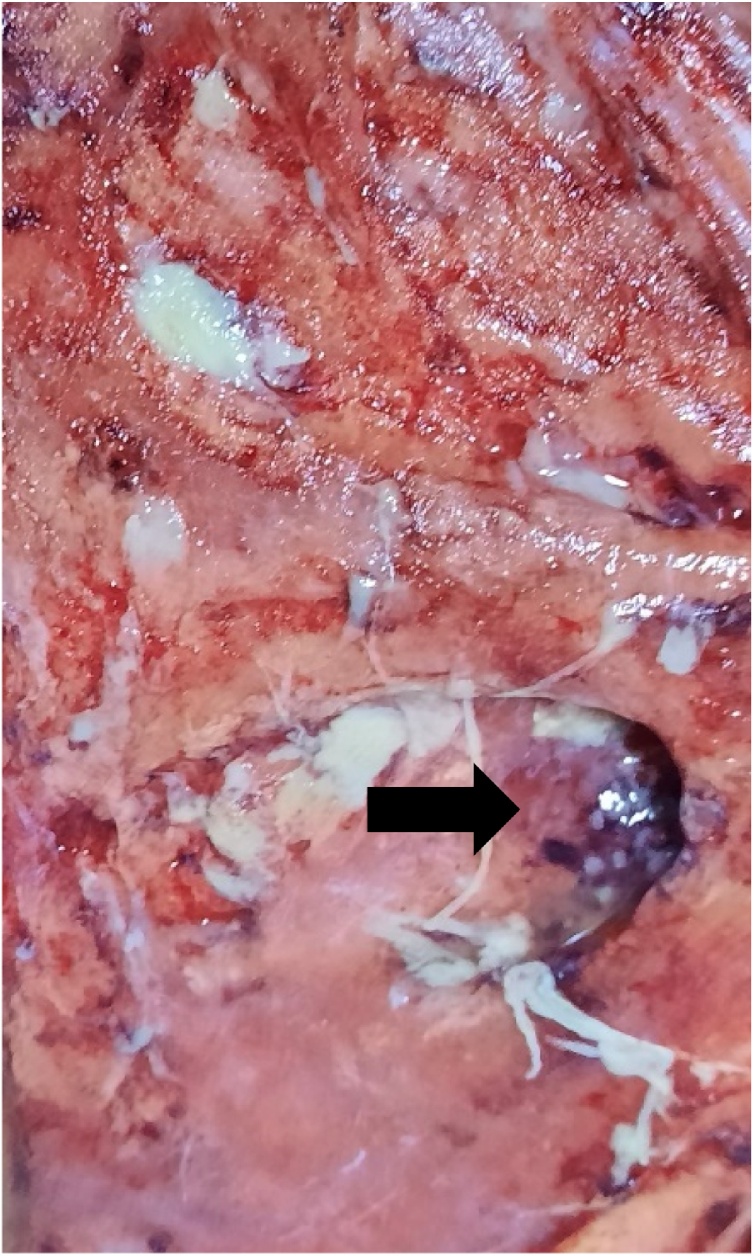
Defect between the abdominal wall and the urinoma used to reach the bladder and perform the biopsy.

A 62-year-old man presented in the emergency department complaining of a recent deterioration of general condition with the appearance of a painless progressive abdominal gangrene without any obstructive lower urinary tract symptoms (LUTS). On medical history, he was diagnosed two months before with a bladder tumour. The patient did not consult because of the Covid-19 pandemic. No history of any trauma or urethral instrumentation was found. On examination, he had a bad general condition and was dehydrated with a blood pressure of 120/75 mm Hg. On abdominal inspection, we noticed a painless large necrotic lesion spread over the anterior wall of the abdomen under the umbilicus with a smell of urine (Fig. 1, Fig. 2). On palpation, there was a renitent mass in the hypogastrium. There were no features suggestive of peritonitis. A urethral catheterization by a Foley catheter failed due probably to anterior urethral stenosis. Basic investigation on admission reported high creatinine levels at 513 umol/l with no electrolytic disorders. Complete blood count showed a decreased haemoglobin level at 6.6 g/dl with thrombocytopenia at 96,000 cmm. A contrast-enhanced abdominal and pelvic computed tomography (CT) was then performed and revealed the presence of a large tumour of the posterior wall of the bladder with bilateral ureteropelvic dilatation, the bladder was distended and perforated on its anterior wall with an extraperitoneal urinoma 10 × 12 cm evoking an extraperitoneal urinary bladder rupture. There was no peritoneal effusion (Fig. 3). The established diagnosis was abdominal gangrene secondary to an extraperitoneal rupture of the urinary bladder. Our attitude was to put a catheter to relieve the urinoma and a suprapubic catheter to drain urine from the bladder and to begin empiric antibiotic therapy. Bilateral nephrostomies were set in order to improve creatinine levels. The patient remained afebrile and stable. After a rapid optimization of the patient’s condition, we carried out excision of all necrotic tissue, we found a 2-cm defect in the anterior bladder wall which was used to perform a biopsy from the tumour (Fig. 4, Fig. 5). The procedure was performed by a urologist and a plastic surgeon with a 20-year experience in gangrene surgery. A cystectomy was difficult to achieve because of the patient’s general condition which did not permit to perform such a surgery. The patient did well without complications postoperatively. After normalization of creatinine levels, a body-CT-scan was performed 12 days after drainage and 3 days postoperatively, it has demonstrated total resolution of the urinoma and showed no metastases. The patient was discharged home on the fifteenth day. Pathologic examination of the biopsy concluded to a massive transitional cell carcinoma with minimal micropapillary differentiation, it did not allow to stage the tumour due to the quality of the pathologic material. In the multidisciplinary consultation meeting, we opted for palliative management by transurethral resection of the bladder tumour (TURB) considering that the tumour is locally advanced and curative cystectomy could not be performed, then palliative chemotherapy should be discussed depending on the course of the disease and the patient's condition.

## 3 Discussion

Urinary bladder rupture is an uncommon occurrence with an incidence reported to be in the region of 1 in 126 000 hospital admissions [1]. It is usually caused by blunt or penetrating trauma or with iatrogenic injury [2,5]. Idiopathic spontaneous ruptures of the urinary bladder reported in the literature are due to ongoing chronic diseases of the urinary bladder (42%) or prolonged urinary retention (35%) [1]. Conditions associated with disorders of bladder wall are bladder diverticulum [1,4], tuberculosis [6,7], chronic/fungal cystitis [8,9], previously repaired bladder perforation [10], irradiation [11], and carcinoma urinary bladder [12]. To our knowledge, it is the first case report of an extraperitoneal bladder rupture related to bladder tumour and complicated by abdominal gangrene which is the most interesting part of this report. In the case of spontaneous bladder rupture, accurate preoperative diagnosis is difficult and often delayed in the absence of a history of trauma or pre-existing bladder disease. In our case, the diagnosis was oriented by the presence of gangrene with urine smell and the history of untreated bladder tumour. Symptoms may be insidious in onset or acute with features of frank peritonitis in case of intraperitoneal rupture [13] and acute renal failure [14]. In the case of bladder rupture with the presence of bladder tumour, the site of the perforation depends on where the tumour was situated as shown from previous studies. The possible pathogenesis of bladder rupture in bladder cancer is the precipitation of perforation on the weakened body wall by the tumour. Although the most frequent location for intraperitoneal perforation was the dome or the posterior wall of the bladder [15]. In this case, the location of perforation was in the anterior wall of the bladder and we think that it was caused by an obstruction of the bladder neck by the tumour. Computed Tomography (CT) scan of the abdomen and urinary cystogram can yield diagnostic results [16]. Combination of CT and cystography is an accurate non-invasive method for assessing bladder pathology especially in patients having a suspension of bladder perforation. In our case, the CT scan was sufficient to establish the diagnosis and begin the treatment. The prognosis of spontaneous bladders rupture due to carcinoma is very poor. Most patients die within months ranging from 10 days to 8 months. The mortality rate can range from 25% to 80% depending up the time of diagnosis [17]. No standardized attitude is recommended but radical cystectomy is usually performed in these cases even if the carcinologic prognosis is not favourable in these cases.

## 4 Conclusion

Patients with extraperitoneal rupture of the urinary bladder usually present with unspecific symptoms. Misdiagnosed, fatal complications may occur such as abdominal wall gangrene as specified in this case report. This is a rare but potentially fatal condition with a mortality rate of more than 80%.

## Conflicts of interest

No conflicts of interest to be declared.

## Funding

No sources of funding to be declared.

## Ethical approval

The study is exempt from ethical approval in our institution.

## Consent

Written informed consent was obtained from the patient for publication of this case report and accompanying images. A copy of the written consent is available for review by the Editor-in-Chief of this journal on request.

## Author contribution

Saadi Mohamed Hafedh: data collection, data analysis or interpretation, writing the paper, references.

Mrad Dali Khaireddine: Paper writing and revision.

Moez Rahoui: data collection, data analysis or interpretation, writing the paper.

Ahmed Sallemi: Paper revision.

Sami Ben Rhouma: Paper and figures revision.

Yassine Nouira: Paper revision.

## Registration of research studies

Not applicable.

## Guarantor

Saadi Mohamed Hafedh is the guarantor of the study and accept full responsibility for the work and/or the conduct of the study, had access to the data and controlled the decision to publish.

## Provenance and peer review

Not commissioned, externally peer-reviewed.
